# Supplementary figures and images for: Effect of Copper Chelators via the TGF-β Signaling Pathway on Glioblastoma Cell Invasion
Source: Molecules. 2022 Dec 13;27(24):8851. doi: 10.3390/molecules27248851 (PMC9784955; doi:10.3390/molecules27248851)

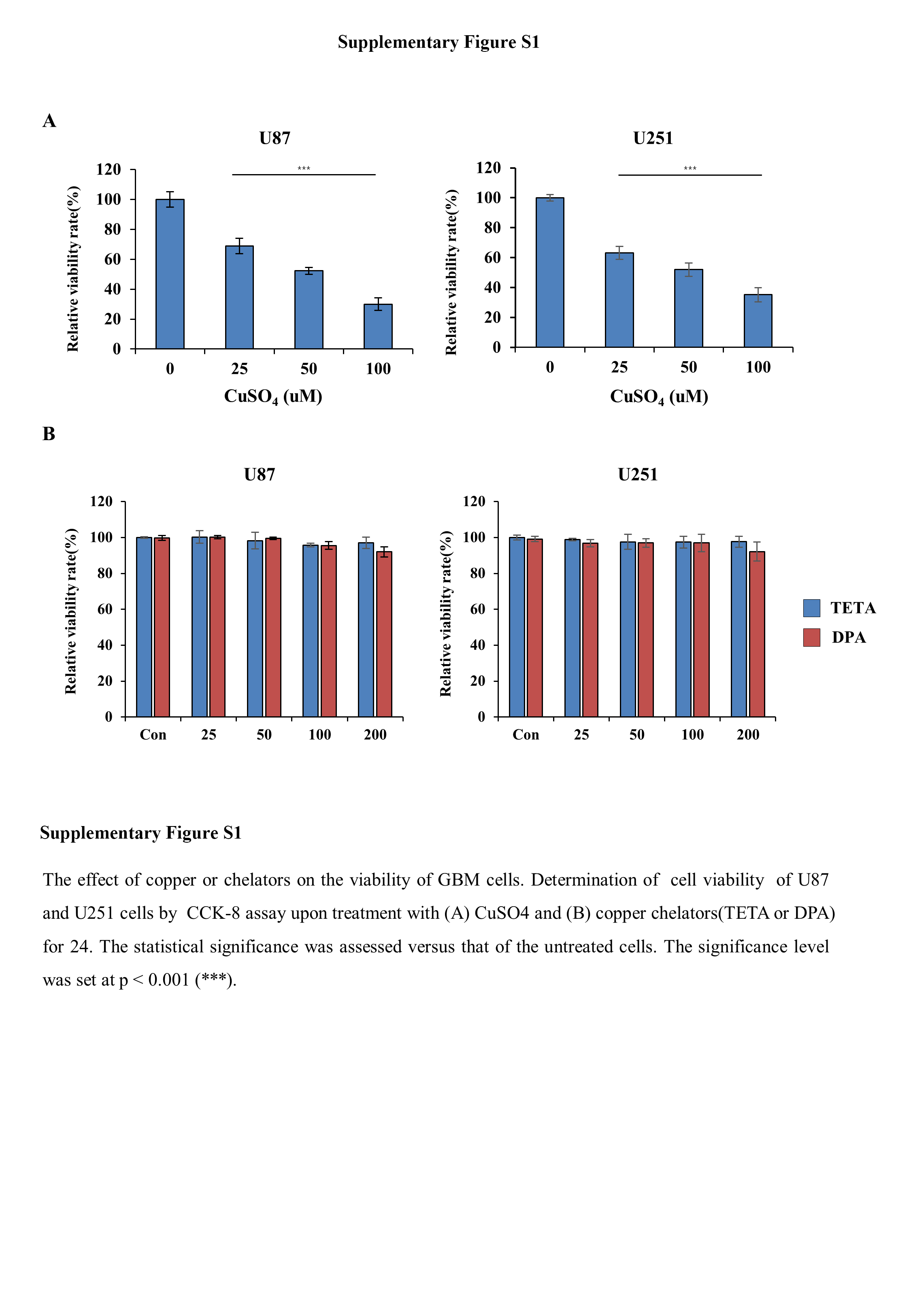

Supplement: Supplementary file 1 [file molecules-27-08851-s001.zip › Supple Figure S1.TIF]

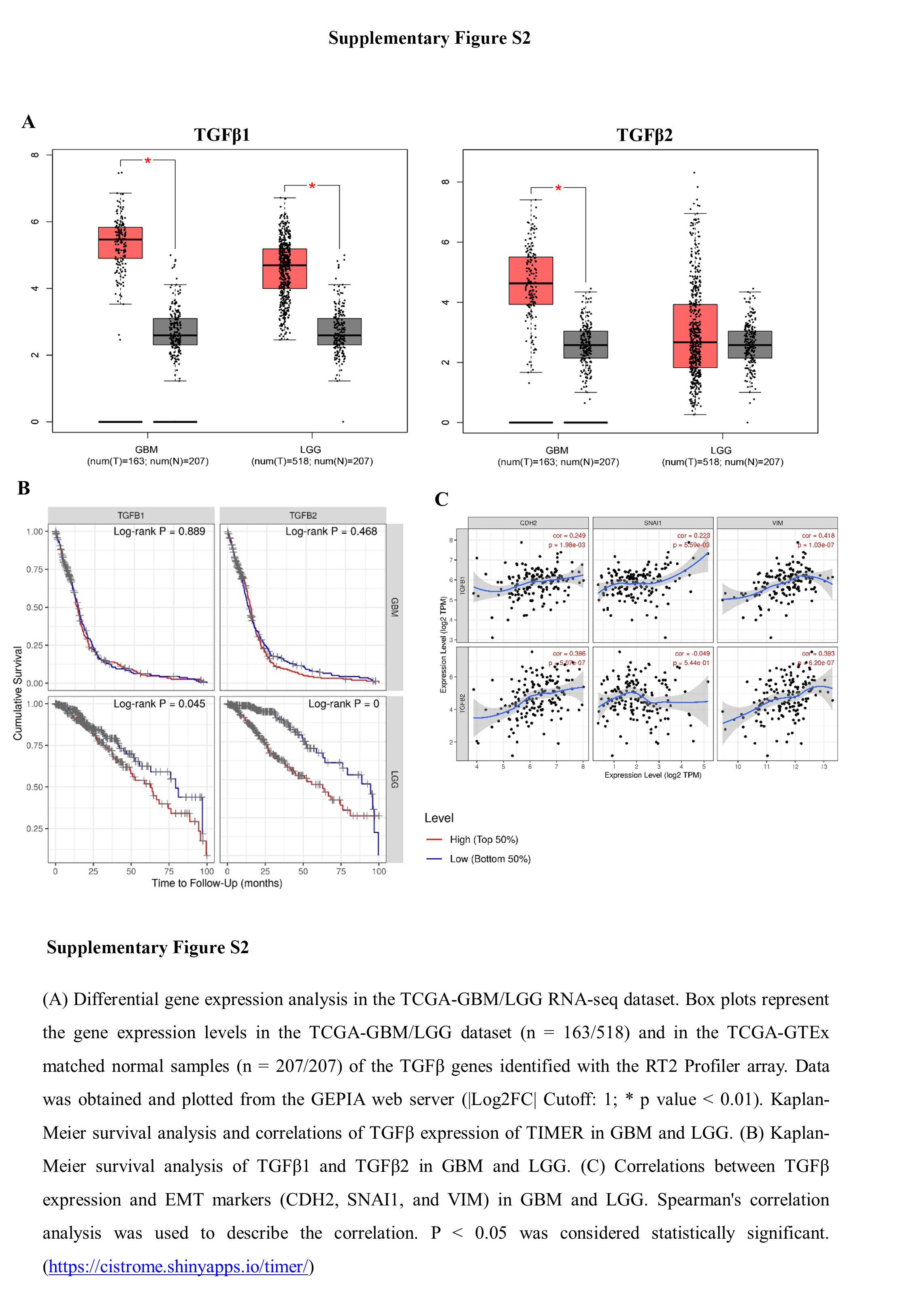

Supplement: Supplementary file 1 [file molecules-27-08851-s001.zip › Supple Figure S2.TIF]

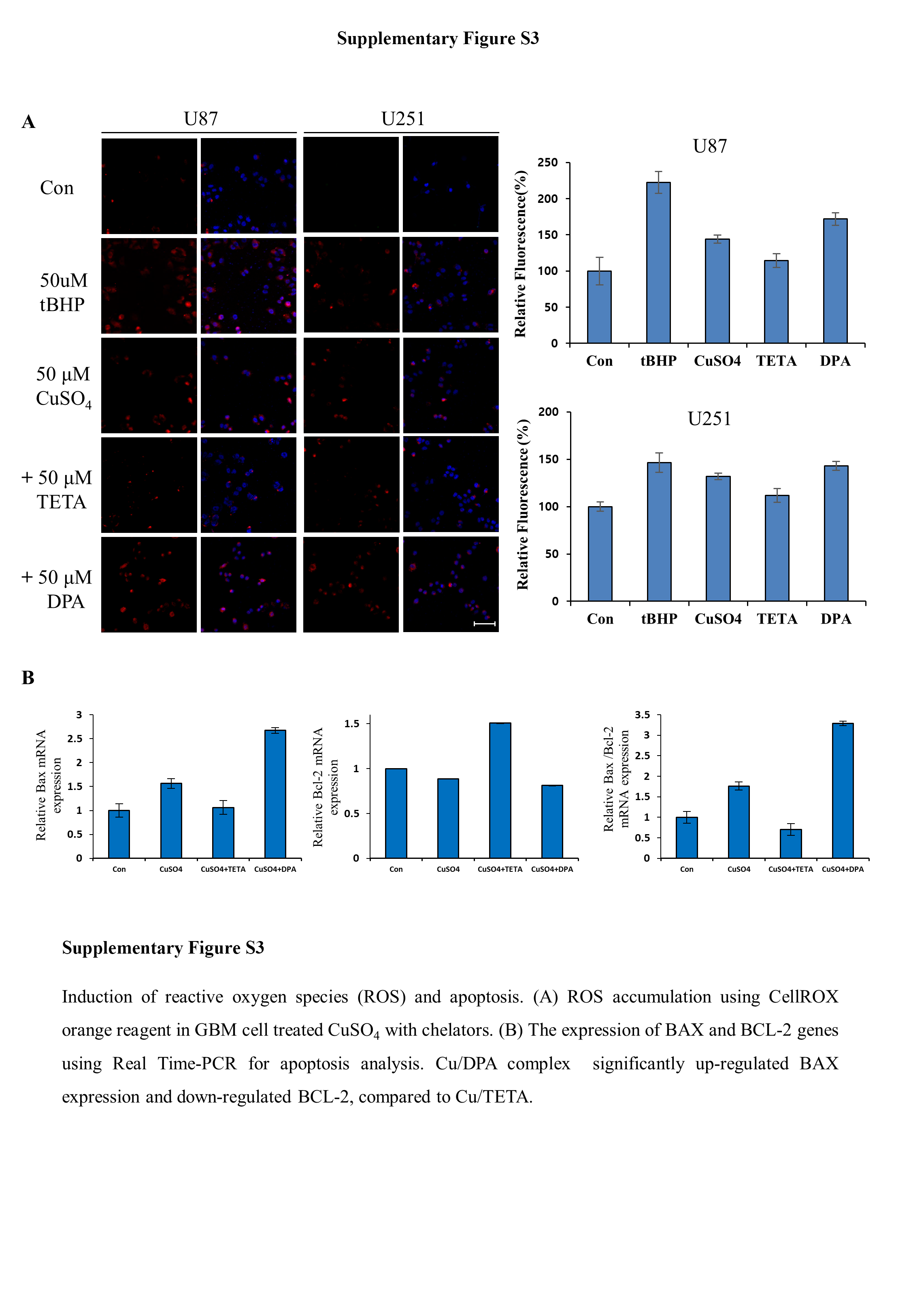

Supplement: Supplementary file 1 [file molecules-27-08851-s001.zip › Supple Figure S3.TIF]

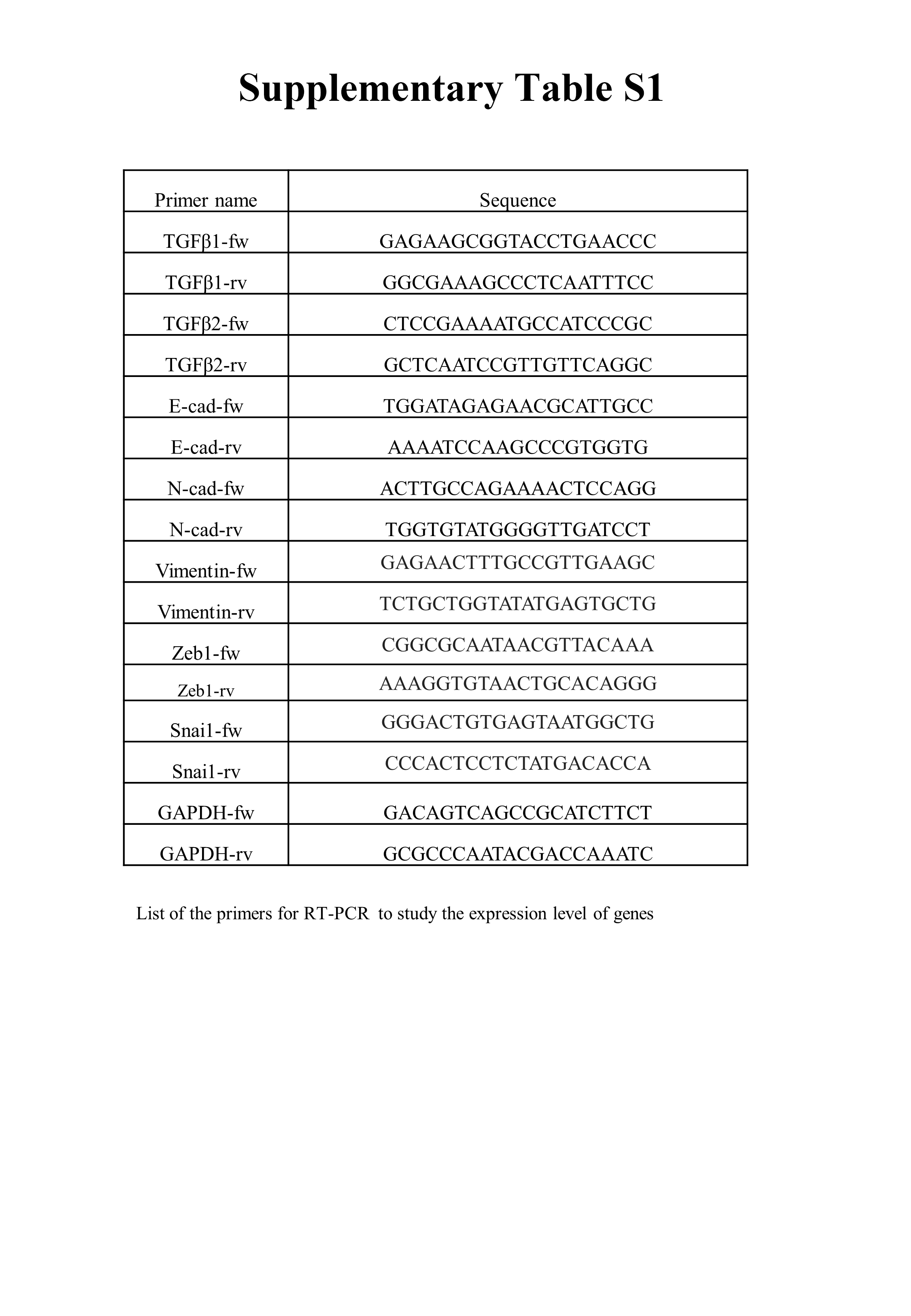

Supplement: Supplementary file 1 [file molecules-27-08851-s001.zip › Supple Table S1.TIF]
